# The Role of Astragaloside IV against Cerebral Ischemia/Reperfusion Injury: Suppression of Apoptosis via Promotion of P62-LC3-Autophagy

**DOI:** 10.3390/molecules24091838

**Published:** 2019-05-13

**Authors:** Yi Zhang, Ying Zhang, Xiao-fei Jin, Xiao-hong Zhou, Xian-hui Dong, Wen-tao Yu, Wei-juan Gao

**Affiliations:** Hebei Key Laboratory of Chinese Medicine Research on Cardio-Cerebrovascular Disease, Hebei University of Chinese Medicine, Shijiazhuang 050200, China; 734910343@163.com (Y.Z.); yingzhang2810@163.com (Y.Z.); jxf1655@163.com (X.-f.J.); zxh19703@163.com (X.-h.Z.); dongxianhuitj@126.com (X.-h.D.); ywtawen@163.com (W.-t.Y.)

**Keywords:** Astragaloside IV, cerebral ischemia/reperfusion injury, autophagy, apoptosis, mechanism

## Abstract

**Background:** Ischemia/reperfusion (I/R) caused by ischemic stroke treatments leads to brain injury, and autophagy plays a role in the pathology. Astragaloside IV is a potential neuroprotectant, but its underlying mechanism on cerebral I/R injury needs to be explored. The objective of this study is to investigate the neuroprotective mechanism of Astragaloside IV against cerebral I/R injury. **Methods:** Middle cerebral artery occlusion method (MCAO) and oxygen and glucose deprivation/reoxygenation (OGD/R) method were used to simulate cerebral I/R injury in Sprague-Dawley (SD) rats and HT22 cells, respectively. The neurological score, 2,3,5-Triphe-nyltetrazolium chloride (TTC) staining, and transmission electron microscope were used to detect cerebral damage in SD rats. Cell viability and cytotoxicity assay were tested in vitro. Fluorescent staining and flow cytometry were applied to detect the level of apoptosis. Western blotting was conducted to examine the expression of proteins associated with autophagy. **Results:** This study found that Astragaloside IV could decrease the neurological score, reduce the infarct volume in the brain, and alleviate cerebral I/R injury in MCAO rats. Astragaloside IV promoted cell viability and balanced Bcl-2 and Bax expression in vitro, reduced the rate of apoptosis, decreased the expression of P62, and increased the expression of LC3II/LC3I in HT22 cells after OGD/R. **Conclusions:** These data suggested that Astragaloside IV plays a neuroprotective role by down-regulating apoptosis by promoting the degree of autophagy.

## 1. Introduction

Ischemic stroke caused by intracerebral arterial embolism can result in local blood supply disturbance leading to high morbidity and mortality. This accounts for 60–80% of all cerebrovascular diseases [1,2]. As one of the common complications of ischemic stroke, cerebral ischemia/reperfusion (I/R) injury frequently occurs during thrombolytic or surgical treatments [3] when blood restores flow. The cellular and molecular mechanisms of cerebral I/R injury involve a variety of pathological processes such as oxidative stress, autophagy, apoptosis, inflammatory reactions, and necrosis; these mechanisms influence the prognosis of cerebral I/R injury [4]. 

**Figure 1:**
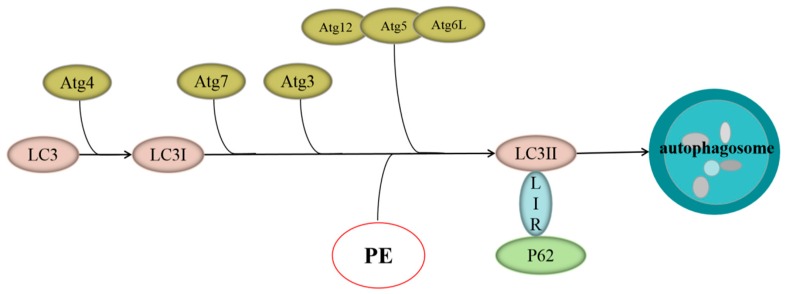
Microtubule-associated protein 1 light chain 3 (LC3)-P62 autophagy signaling pathway.

Recently, researchers discovered that autophagy played a key role in these pathological processes during cerebral I/R injury [5,6,7,8]. Autophagy is a highly conservative evolutionary process that regulates the quantity and quality of organelles and proteins via lysosomal systems to maintain cellular health. It is activated during ischemia to generate energy and promote cell survival [9]. Autophagy begins with the formation of autophagosomes that can be determined by visualization of the microtubule-associated protein 1 light chain 3 (LC3) protein because it is required for autophagosome maturation. The C-terminal polypeptide of LC3 is cleaved by ATG4 protease to expose a glycine residue and generate LC3I. The LC3I is then covalently conjugated to phosphatidylethanolamine (PE) by interacting with ATG7, ATG3, and the ATG12-ATG5-ATG16L complex to generate LC3II. LC3II protein levels serve as a read-out of an autophagosome number, and detecting the conversion of LC3I to LC3II (LC3II/LC3I) is indicative of autophagic activity [10]. Several specific substrates are efficiently degraded by autophagy and the well-characterized receptor is P62 (also known as SQSTM1/sequestome 1). The P62 binds directly to LC3 via a short LC3 interaction region (LIR), and P62 is a vital indicator of autophagic flux. It selectively incorporates into autophagosomes by directly binding to the LC3 on autophagic membranes for subsequent degradation in autolysosomes [11,12,13] (Figure 1). 

Recent studies have shown that several molecules required for autophagy also regulate apoptosis. If P62-dependent recruitment of necrosome to autophagy machinery is blocked, then the mechanism of cell death switches to apoptosis [14]. Additionally, LC3 has been reported to have apoptotic function. Hence, the established relationship between autophagy and apoptosis are closely related to LC3 and P62 signals. Oxidative stress is caused by a large amount of reactive oxygen species, free radicals, etc. after cerebral I/R. It is one of causes of autophagy [15].

Apoptosis is an important mechanism of secondary damage in brain tissue after cerebral I/R injury [16]. It is characterized by cell shrinkage, chromatin condensation, DNA degradation and fragmentation, and cell division into apoptotic bodies, following by phagocyte phagocytosis and degradation [17]. Studies have confirmed that cerebral ischemia leads to Bax (pro-apoptotic protein) translocates to the outer mitochondrial membrane and increases the permeability of mitochondrial outer membrane triggering the intrinsic apoptotic pathway [18,19]. Cytochrome C is then released from mitochondria into the cytoplasm because the mitochondrial outer membrane permeability increases thereby activating the Caspase-3 cascade ultimately leading to DNA degradation and eventually leading to brain injury after cerebral ischemia [20,21]. In this process, Bcl-2 (anti-apoptotic protein) inhibits apoptosis by stabilizing mitochondrial membrane potential and preventing cytochrome C release. Bcl-2 and Bax proteins are key apoptosis regulators [22], and their ratio is an important indicator in determining whether apoptosis occurs or not [23].

Our previous research revealed that BuyangHuanwu decoction (BYHWD), a traditional Chinese medicine prescription for treating ischemic stroke recorded in the book of “Yilin Gaicuo (Correction on the errors of medical works)” two hundred years ago, showed significant neuroprotective effects [24,25,26]. BuyangHuanwu decoction could enhance hippocampal neuronal plasticity and increase ERK2, CaMKIIβ mRNA and protein expression during cerebral I/R injury [27]. *Astragalus membranaceus*, a traditional Chinese Qi-tonifying herb [28], is the main drug of BYHWD [29] and has been used in the treatment of ischemic stroke for thousands of years in China [30,31,32].

**Figure 2:**
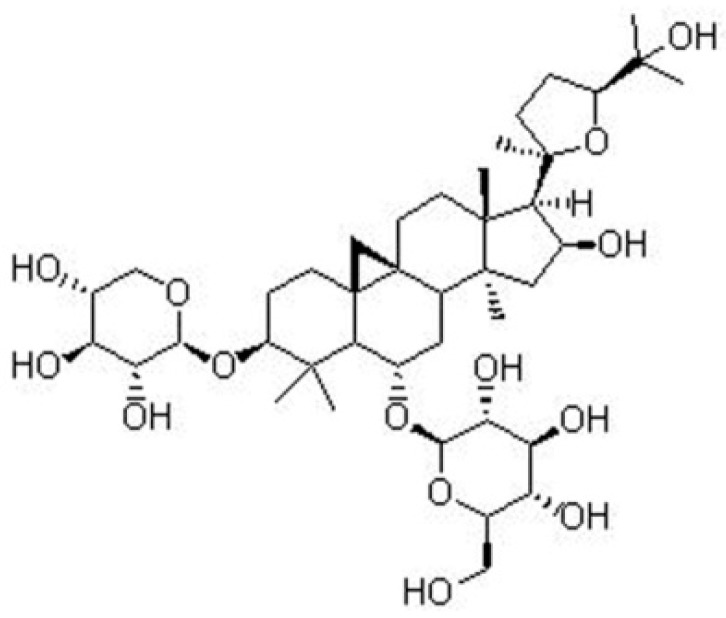
Chemical structures of Astragaloside IV.

Astragaloside IV, 3-O-beta-d-xylopyranosyl-6-O-beta-d-glucopyranosyl-cycloastragenol, is a purified small molecular weight saponin isolated from the dried plant roots of *Astragalus membranaceus*. Its molecular formula is C_14_H_68_O_14_ (Figure 2). Astragaloside IV serves as a quality-control marker component of *Astragalus membranaceus* in the Chinese Pharmacopoeia and has been widely used as a neuroprotective agent [33]. Studies have shown that Astragaloside IV has biological activities include as an anti-oxidant, anti-inflammatory, anti-virus, anti-aging, and anti-platelet aggregation. It can reduce cerebral I/R injury by inhibiting apoptosis and improving energy metabolism [34]. However, there was no report on Astragaloside IV’s neuroprotective effects by regulating autophagy to inhibit apoptosis. To test the hypothesis that Astragaloside IV plays a neuroprotective role by inhibiting apoptosis via regulating autophagy, the effect of Astragaloside IV on apoptosis was observed after autophagy was intervened by autophagy inhibitor-3-methyladenine (3ma) and autophagy activator-rapamycin (Rapa) in this study.

In this study, Astragaloside IV relieves cerebral I/R injury in vivo and in vitro. The effect of Astragaloside IV on apoptosis and autophagy in cerebral I/R injury was also investigated. Finally, the relationship between apoptosis and autophagy in the context of cerebral I/R injury was verified.

## 2. Results

### 2.1. Astragaloside IV Inhibits Cerebral Damage following Ischemia/Reperfusion Injury

**Figure 3:**
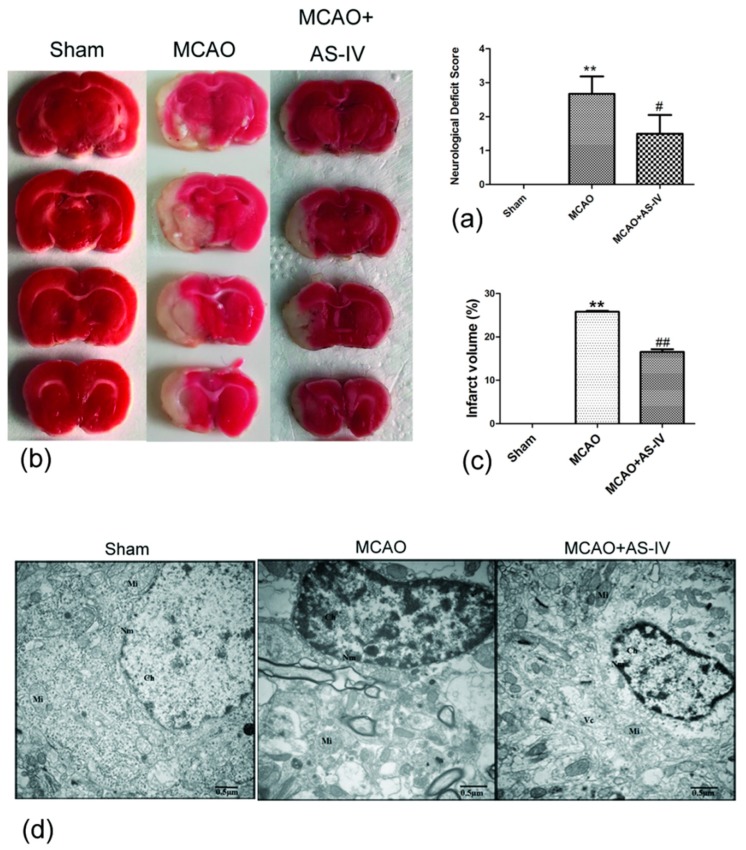
Neuroprotective effect of Astragaloside IV in rats after middle cerebral artery occlusion method (MCAO). (**a**) The neurological scores of rats (measured at 24 h after MCAO) (means ± SD, *n* = 6, ** *p* < 0.01 vs. Sham, ^#^ *p* < 0.05 vs. MCAO); (**b**,**c**) representative 2,3,5-Triphe-nyltetrazolium chloride (TTC) stained serial coronal brain sections (measured at 24 h after MCAO) (means ± SD, *n* = 3, ** *p* < 0.01 vs. sham, ^##^ *p* < 0.01 vs. MCAO); (**d**) observation of ultrastructure of neurons by transmission electron microscope. (scale bar = 0.5 µm); Nm: Nuclear membrane; Ch: Chromatin; Mi: Mitochondria; Vc: Vacuole.

To test the neuroprotective effects of Astragaloside IV, neurological score and 2,3,5-Triphe-nyltetrazolium chloride (TTC) staining were performed on middle cerebral artery occlusion method (MCAO) rats. Rats were administrated Astragaloside IV (20 mg/kg) for 24 h after reperfusion, and neurological deficits were observed. The Zea Longa Neurological Score can effectively evaluate neurological deficits in MCAO rats [35], and there were no neurological deficits in the sham group (score = 0), but the MCAO group increased significantly (score in MCAO group, 2.67 ± 0.52; *p* < 0.01 vs. Sham). Astragaloside IV could significantly decrease the neurological score in MCAO rats (score in MCAO + Astragaloside IV (AS-IV) group, 1.50 ± 0.55; *p* < 0.01 vs. MCAO) (Figure 3a).

The TTC staining showed that rats in the sham group showed no evidence for cerebral infraction, but the infarct volume in the MCAO group was 25.79 ± 0.21% (*p* < 0.01 vs. sham group). Astragaloside IV treatment reduced the volume of cerebral infraction (the infarct volume in MCAO + AS-IV group was 16.54 ± 0.62%, *p* < 0.01 vs. MCAO group) (Figure 3b,c).

We further observed the ultrastructure of neurons via transmission electron microscopy. In the sham group, the nuclear membrane is clear and complete, and even chromatin and normal mitochondria could be seen. However, the neurons had ruptured with a dissolved nuclear membrane. The chromatin had condensed with many vacuoles and swollen mitochondria in the MCAO group. In the MCAO + AS-IV group, the nuclear membrane was smoother and more complete with minimal mitochondrial swelling (Figure 3d). These results indicated that Astragaloside IV can effectively improve the behavior of MCAO rats and alleviate I/R injury.

### 2.2. Astragaloside IV Increased the Viability of HT22 Cells after OGD/R

**Figure 4:**
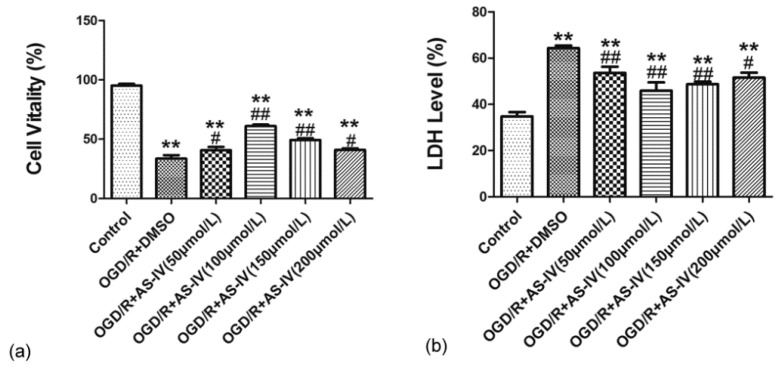
Neuroprotective effect of Astragaloside IV in HT22 cells after oxygen and glucose deprivation/reoxygenation (OGD/R). (**a**) Viability of HT22 cells treated with different concentrations of Astragaloside IV after OGD/R examined by Cell Counting Kit-8 (CCK-8) (means ± SD, *n* = 6, ** *p* < 0.01 vs. control and ^#^ *p* < 0.05, ^##^ *p* < 0.01 vs. OGD/R + dimethyl sulfoxide (DMSO)); (**b**) lactate dehydrogenase (LDH) level of HT22 cells treated by different concentrations of Astragaloside IV after OGD/R examined by LDH (means ± SD, *n* = 6, ** *p* < 0.01 vs. control and ^#^ *p* < 0.05, ^##^ *p* < 0.01 vs. OGD/R + DMSO).

We further observed the effect of Astragaloside IV on OGD/R HT22 cells after confirming its neuroprotective effects on MCAO rats. The neuroprotective effects of different doses of Astragaloside IV (50, 100, 150 and 200 µmol/L) were detected by Cell Counting Kit-8 (CCK-8) and lactate dehydrogenase (LDH) in HT22 cells after OGD/R. The CCK-8 test result indicated that cell viability significantly decreased after OGD/R (cell viability in the OGD/R + dimethyl sulfoxide (DMSO) group and in 50, 100, 150, and 200 µmol/L Astragaloside IV groups were 33.75 ± 2.86%, 40.71 ± 2.73%, 61.12 ± 1.19%, 49.29 ± 1.55%, and 40.89 ± 1.18%, respectively; *p* < 0.01 vs. control group). Different concentrations of Astragaloside IV could improve cell viability after OGD/R (*p* < 0.05 vs. OGD/R + DMSO group) (Figure 4a).

The LDH release test result was consistent with the CCK-8 test. The LDH level significantly increased in the OGD/R + DMSO group and different concentrations of Astragaloside IV groups compared with the control group (LDH level in the OGD/R + DMSO group and in 50, 100, 150, and 200 µmol/L Astragaloside IV groups were 64.30 ± 1.13%, 53.60 ± 1.04%, 45.89 ± 3.61%, 48.72 ± 1.23%, and 51.56 ± 2.12%, respectively; *p* < 0.01 vs. control group), and different concentrations of Astragaloside IV significantly reduced the LDH level in OGD/R HT22 cells (*p* < 0.05 vs. OGD/R + DMSO group) (Figure 4b). This indicated that Astragaloside IV has neuroprotective effect on OGD/R HT22 cells, and Astragaloside IV at 100 µmol/L was used in the following experiments.

### 2.3. Astragaloside IV Reduced Apoptosis in OGD/R HT22 Cells

**Figure 5:**
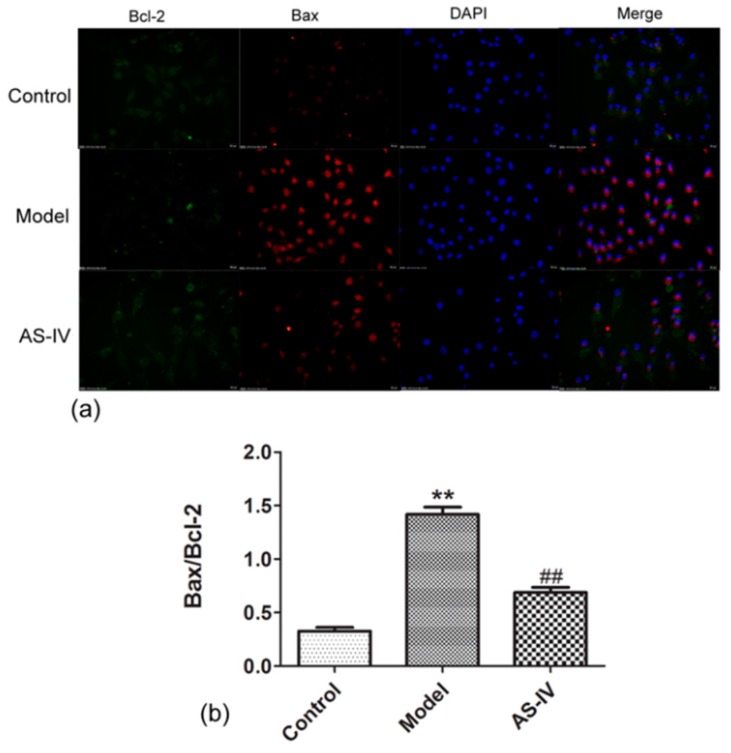
Astragaloside IV reduced Bax/Bcl-2 in HT22 cells after OGD/R immunofluorescence staining for Bax and Bcl-2 of HT22 cells after OGD/R (means ± SD, *n* = 3. ** *p* < 0.01 vs. control and ^##^ *p* < 0.01 vs. model). (scale bar = 20 µm) Fluorescent images: Green, Bcl-2; red, Bax; and blue, DAPI. (**a**) Representative photographs obtained from each group. (**b**) Quantitative assessment of Bax/Bcl-2.

To observe the effect of anti-apoptosis of Astragaloside IV in cerebral I/R injury, Bax/Bcl-2 was detected by immunofluorescence staining, and the apoptosis rate was tested via flow cytometry in HT22 cells. The immunofluorescence staining showed that Bax/Bcl-2 was low in the control group (Bax/Bcl-2 in control group, 0.33 ± 0.03), but it significantly increased in HT22 cells after OGD/R (Bax/Bcl-2 in model, 1.42 ± 0.07, *p* < 0.01 vs. sham group). Astragaloside IV significantly reduced Bax/Bcl-2 in OGD/R HT22 cells (Bax/Bcl-2 in AS-IV group, 0.69 ± 0.05; *p* < 0.01 vs. MCAO group; Figure 5).

**Figure 6:**
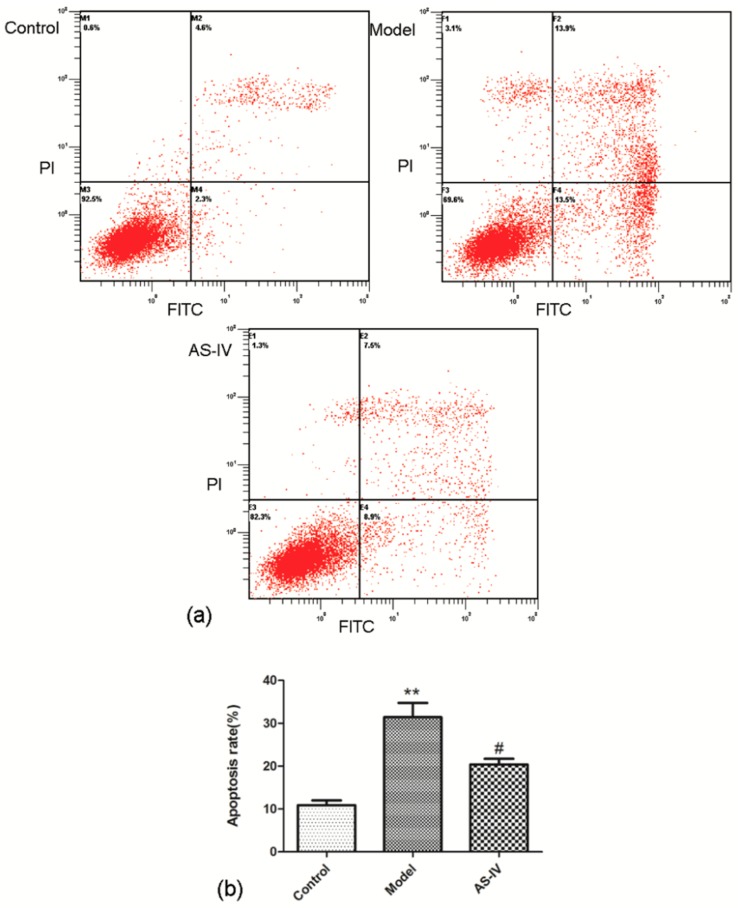
Anti-apoptosis effect of Astragaloside IV in HT22 cells after OGD/R. The changes in the apoptosis rates in the HT22 cells by OGD/R with Astragaloside IV treatment examined by a flow cytometry with Annexin V-FITC/PI staining (means ± SD, *n* = 3, ** *p* < 0.01 vs. control and ^#^ *p* < 0.05 vs. model). (**a**) Representative photographs obtained from each group. (**b**) Quantitative assessment of apoptotic cell death.

The flow cytometry data showed that the apoptosis rate significantly increased in HT22 cells after OGD/R (apoptosis rate in model group, 31.43 ± 3.33, *p* < 0.01 vs. control group) and Astragaloside IV significant decreased apoptosis rate in OGD/R HT22 cells (apoptosis rate in AS-IV group, 20.37 ± 1.36; *p* < 0.05 vs. model group; Figure 6).

### 2.4. Astragaloside IV Enhanced Autophagy on HT22 Cells after OGD/R

**Figure 7:**
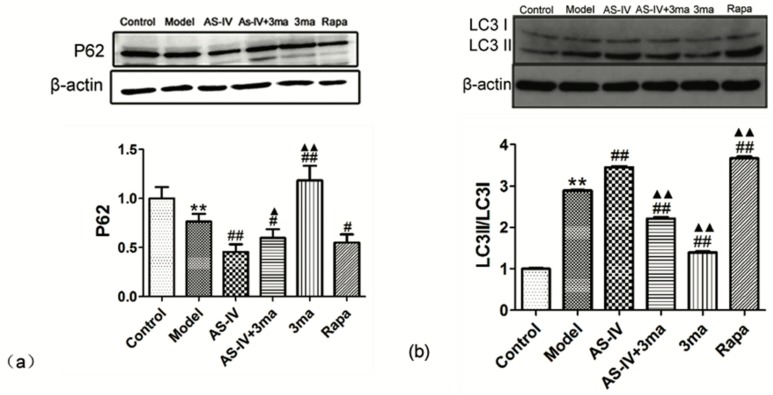
Enhancement of autophagy by Astragaloside IV after OGD/R. (**a**) The expression of P62 in HT22 cells after OGD/R with different treatments (** *p* < 0.01 vs. control, ^#^ *p* < 0.05, ^##^ *p* < 0.01 vs. model and ^▲^ *p* < 0.05, ^▲▲^ *p* < 0.01 vs. AS-IV); (**b**) LC3II/LC3I in HT22 cells after OGD/R with different treatments (** *p* < 0.01 vs. control, ^##^ *p* < 0.01 vs. model and ^▲▲^ *p* < 0.01 vs. AS-IV). Representative photographs obtained from each group (above) and statistical analysis from all cells in each group (below).

The effect of Astragaloside IV on autophagy in cerebral I/R injury was also explored. We treated OGD/R HT22 cells with Astragaloside IV, Astragaloside IV + 3ma (a phosphatidylinositol-3-kinase inhibitors that blocks autophagy), 3ma, or Rapa (Rapa induces autophagy by inhibiting mTOR). Western blot results showed significant changes in the expression of P62 in different groups. The expression of P62 decreased significantly in HT22 cells after OGD/R (the expression of P62 in model group, 0.76 ± 0.08; *p* < 0.01 vs. control group). Versus the model group, the expression of P62 in AS-IV, AS-IV + 3ma, and Rapa groups significantly decreased (the expression of P62 in AS-IV, AS-IV + 3ma, and Rapa groups were 0.45 ± 0.08, 0.60 ± 0.09, and 0.55 ± 0.08, respectively; *p* < 0.05 vs. model group). The values increased significantly in the 3ma group (the expression of P62 in 3ma group, 1.18 ± 0.15; *p* < 0.01 vs. model group). Compared with the AS-IV group, the expression of P62 in the AS-IV + 3ma group and the 3ma group was significantly increased (*p* < 0.05 vs. AS-IV group). There was no significant difference between the Rapa group and the AS-IV group (Figure 7a).

LC3 is an important autophagy biomarker, and LC3II/LC3I increases when autophagy occurs in cells. Western blotting showed that OGD/R led to a significant increase in LC3II/LC3I (LC3II/LC3I in model group, 2.89 ± 0.01; *p* < 0.01 vs. control group). Both Astragaloside IV and Rapa could further improve LC3II/LC3I (LC3II/LC3I in AS-IV and Rapa groups were 3.45 ± 0.02 and 3.67 ± 0.04, respectively; *p* < 0.01 vs. model group). The autophagy inhibitor-3ma significantly downregulated LC3II/LC3I and inhibited the effect of Astragaloside IV (LC3II/LC3I in AS-IV + 3ma and 3ma groups were 2.21 ± 0.03, 1.40 ± 0.02, respectively; *p* < 0.01 vs. model group). Compared with the AS-IV group, LC3II/LC3I in the AS-IV + 3ma group and the 3ma group was significantly decreased (*p* < 0.01 vs. AS-IV group), while the LC3II/LC3I significantly increased in Rapa group (*p* < 0.01 vs. AS-IV group) (Figure 7b). This indicated that Astragaloside IV enhances autophagy.

### 2.5. Astragaloside IV Inhibited Apoptosis on HT22 Cells after OGD/R by Activating Autophagy

**Figure 8:**
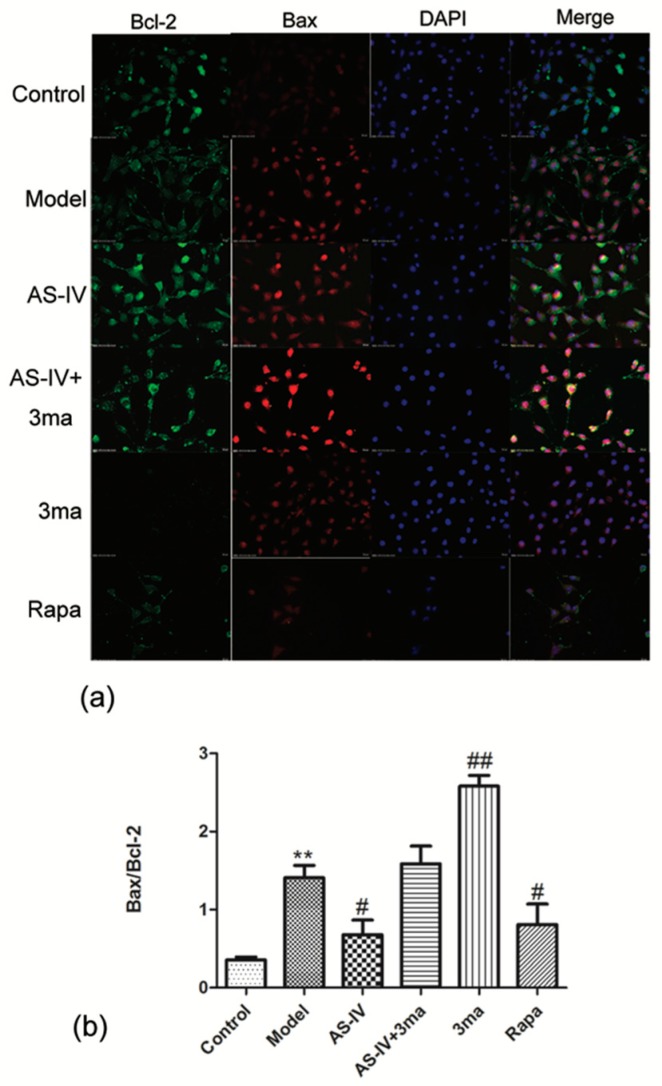
Astragaloside IV reduced Bax/Bcl-2 by activating autophagy after OGD/R immunofluorescence staining for Bax and Bcl-2 of HT22 cells by OGD/R (means ± SD, *n* = 3. ** *p* < 0.01 vs. control and ^#^ *p* < 0.05, ^##^ *p* < 0.01 vs. model; scale bar = 20 µm). Fluorescent images: Green, Bcl-2; red, Bax; and blue, DAPI. (**a**) Representative photographs obtained from each group. (**b**) Quantitative assessment of Bax/Bcl-2.

Finally, we explored the relationship between apoptosis and autophagy in OGD/R HT22 cells and observed the effect of Astragaloside IV on apoptosis in OGD/R cells after autophagy was inhibited by the autophagy inhibitor 3ma. First, changes of Bax/Bcl-2 in HT22 cells after different treatments were observed. The ratio of Bax and Bcl-2 in HT22 cells was significantly increased by OGD/R (Bax/Bcl-2 in the model group (1.41 ± 0.16; *p* < 0.01 vs. control group). Both Astragaloside IV and Rapa could reduce the ratio of Bax and Bcl-2 (Bax/Bcl-2 in Astragaloside IV and Rapa treated samples were 0.68 ± 0.19 and 0.81 ± 0.26, respectively; *p* < 0.05 vs. model group). This result may be related to the activation of autophagy by AS-IV and Rapa. Bax/Bcl-2 was further elevated in OGD/R HT22 cells after treatment with 3ma (Bax/Bcl-2 with 3ma was 2.58 ± 0.14; *p* < 0.01 vs. model group). The effect of anti-apoptosis of Astragaloside IV was inhibited by 3ma (Bax/Bcl-2 in AS-IV + 3ma group, 1.59 ± 0.23, *p* > 0.05 vs. model group; Figure 8).

**Figure 9:**
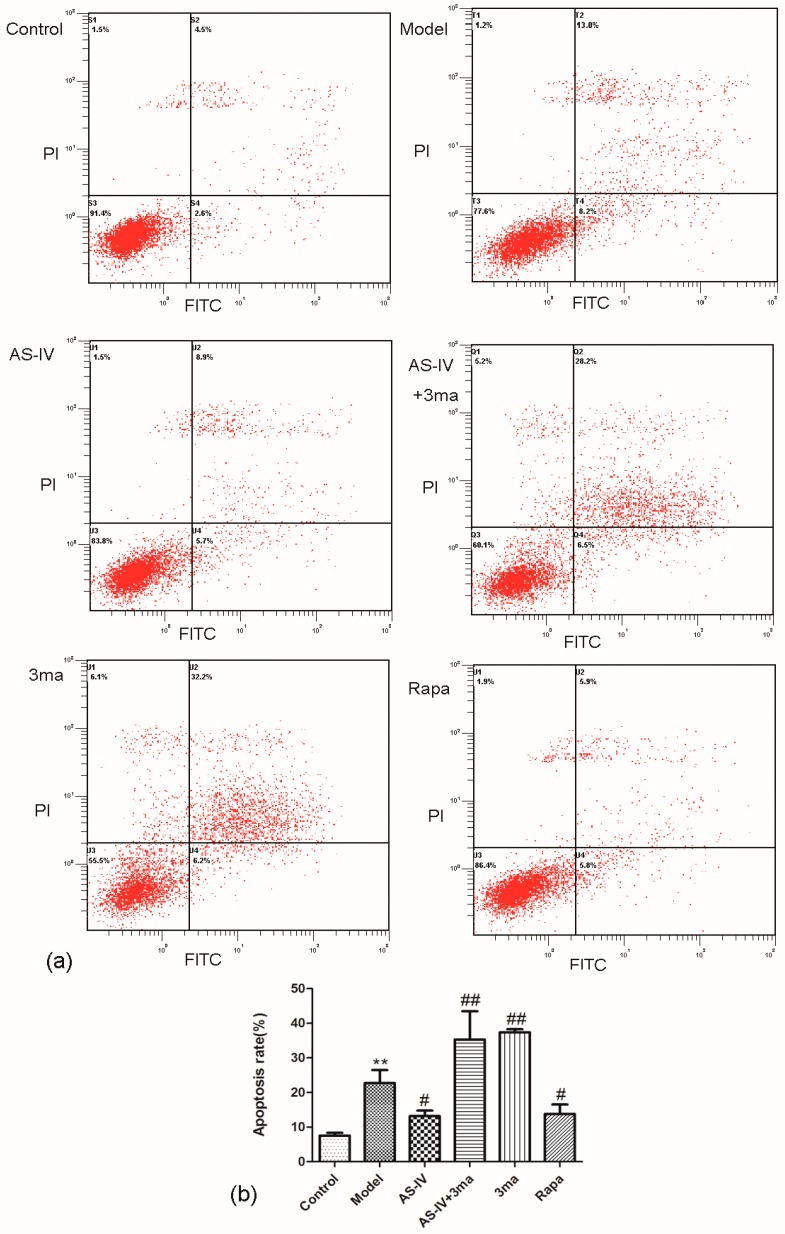
Astragaloside IV decreased the apoptosis rate by activating autophagy after OGD/R. The changes of the apoptotic rates in the HT22 cells by OGD/R with different treatments examined via flow cytometry with Annexin V-FITC/PI staining (means ± SD, *n* = 3. ** *p* < 0.01 vs. control and ^#^ *p* < 0.05, ^##^ *p* < 0.01 vs. model). (**a**) Representative photographs obtained from each group. (**b**) Quantitative assessment of apoptotic cell death.

We also directly observed the apoptosis of OGD/R HT22 cells by flow cytometry. The flow cytometry data showed that the apoptosis rate in HT22 cells significant increased after OGD/R (apoptosis rate in model group, 22.73 ± 3.74; *p* < 0.01 vs. control group). Astragaloside IV and Rapa could reduce the apoptosis rate in OGD/R HT22 cells by activating autophagy (apoptosis rates in AS-IV and Rapa groups were 13.20 ± 1.56 and 13.80 ± 2.70, respectively; *p* < 0.05 vs. model group). The autophagy inhibitor 3ma significantly increased apoptosis rate and inhibited the effect of Astragaloside IV on apoptosis rate (apoptosis rate in AS-IV + 3ma and 3ma groups were 35.30 ± 8.16 and 37.40 ± 0.89, respectively; *p* < 0.01 vs. model group; Figure 9). These results verified that Astragaloside IV reduced apoptosis by improving autophagy. The anti-apoptosis effects of Astragaloside IV were inhibited by 3ma.

## 3. Discussion

This study showed that Astragaloside IV protected the brain from ischemia/reperfusion injury in vivo and protected neurons from OGD/R-induced injury in vitro. The mechanism of efficacy of Astragaloside IV were found to down-regulated apoptosis included P62-LC3-autophagy during OGD/R injury. An accumulating body of evidence has confirmed that apoptosis—an important mechanism of secondary injury—will significantly increase during cerebral I/R injury [36,37,38]. As an important neuroprotective agent, Astragaloside IV has a significant effect of anti-apoptosis [39,40] and alleviates cerebral I/R injury by suppressing apoptosis expression [41]. Here, we found that OGD/R treatment of HT22 cells resulted in elevated apoptosis, and Astragaloside IV could effectively reduce apoptosis expression (Figure 5, Figure 6, Figure 8 and Figure 9) consistent with prior reports [36,37,38,39,40,41].

The Bcl-2 family plays a key role in regulating the initiation of mitochondrial pathways of apoptosis [40]. Bcl-2 and Bax are in equilibrium under normal circumstances. Bax translocates to the mitochondria and enhances mitochondrial membrane permeability when stimulated by ischemia or other stimuli. This can lead to the release of cytochrome C and apoptosis-inducing factor (AIF), which in turn triggers apoptosis [42]. However, Bcl-2 inhibits cell apoptosis by stabilizing the mitochondrial membrane potential and preventing cytochrome C release [43]. Changes in the ratio of Bax to Bcl-2 determine neuronal apoptosis and survival. HT22 cell is an immortalized mouse hippocampal cell line subcloned from the HT-4 cell line. Because it has some characteristics of hippocampal neurons and has the advantage of easy culture, it is currently widely used in the study of neurodegenerative diseases and strokes [44,45]. Therefore, we used HT22 cells as in vitro subjects to observe the effects of OGD/R and Astragaloside IV on apoptosis of it. Here, Bax/Bcl-2 increased significantly in OGD/R HT22 cells, which indicated that apoptosis was triggered and induced in cerebral I/R injury. While Astragaloside IV significantly reduced Bax/Bcl-2 level in OGD/R HT22 cells and served as an anti-apoptosis element in vitro (Figure 5 and Figure 7). These results are consistent with the results of Chumboatong [46], Wicha [47], and Zhang [36]. Thus, we were curious if Astragaloside IV reduces apoptosis by increasing the level of autophagy.

Autophagy can be induced via nutrient depletion or I/R injury [48,49]. Moderating the activation of autophagy in ischemic conditions not only provides energy by degrading proteins but also protect cells by degrading damaged proteins and synthesizing new proteins [50,51]. Silvia et al. found that autophagy is enhanced in neuronal cells after neonatal hypoxia-ischemia and represents a potential protective mechanism in cerebral injury [49]. Sheng et al. found that oxygen–glucose deprivation could lead to autophagy in PC12 cells, and inhibition of autophagy resulted in decreased cell viability and injury aggravation [52]. Therefore, we observed autophagy expression in HT22 cells after OGD/R and explored the effectiveness of Astragaloside IV on autophagy. Autophagy is a biological process involving a series of autophagy-related proteins including LC3I and LC3II. LC3II increases with autophagosome membranes formation [53]. P62 is a bridge connecting LC3 protein with the ubiquitinated substrate to be degraded. P62will be degraded when autophagy occurs [54]. We found that OGD/R could increase autophagy via accumulation of LC3II/LC3I and decreased P62 expression. Moreover, Astragaloside IV could further enhance autophagy by increasing the LC3II/LC3I ratio and inhibiting the pP62 expression. Liu [55], Wang [56] and Qu [57] confirmed that Astragaloside IV could activate autophagy in human degenerative chondrocytes and renal disease, respectively. We found that Astragaloside IV can activate autophagy in HT22 cells after OGD/R, it is consistent with the results of the above studies [55,56,57] about the effects of Astragaloside IV on autophagy.

**Figure 10:**
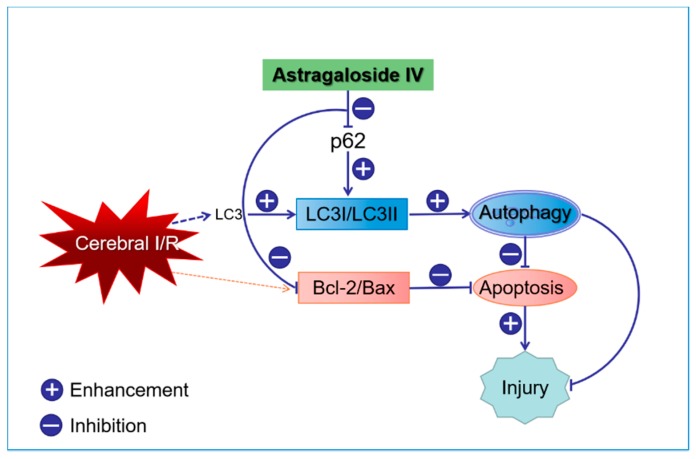
The neuroprotective mechanisms of Astragaloside IV against cerebral ischemia/reperfusion (I/R) injury.

As an evolutionary and conservative mechanism that determines cell fate, there is an intricate relationship between apoptosis and autophagy: Activation of autophagy after cerebral ischemia can inhibit cell apoptosis and relieve brain damage. Thus, we hypothesized that the underlying mechanism of anti-apoptosis of Astragaloside IV may be due to activation of autophagy. Changes in apoptosis by administering Astragaloside IV, an autophagy inhibitor, or an autophagy activator were seen. Hu [58] and Wu [2] demonstrated that activation of autophagy can inhibit apoptosis in the Parkinson’s model and stroke’s models. Similar to Hu [58] and Wu [2], we confirmed that the apoptosis level in OGD/R cells increased after inhibiting autophagy; apoptosis declined after activating autophagy process. Astragaloside IV could further enhance the level of autophagy characterized by reducing the level of P62 expression and elevating LC3II/LC3I ratio. This inhibited apoptosis in HT22 cells after OGD/R. However, the anti-apoptosis effect of Astragaloside IV was inhibited after administration of an autophagy inhibitor (Figure 10).

In this study, we investigated the effects of Astragaloside IV on apoptosis and autophagy using immortalized neurons-HT22 cells. In future studies, we will use the primarily cultured hippocampal neurons and brain samples of the MCAO rats to further investigate the effects of Astragaloside IV on apoptosis and autophagy in cerebral I/R injury, and explored other pathways which Astragaloside IV may undergo neuroprotective activities.

## 4. Materials and Methods

### 4.1. Chemicals and Reagents

Dulbecco’s Modified Eagle’s Medium (DMEM) and Trypsin-EDTA were provided by Gibco (Grand Island, NY, USA). Fetal bovine serum (FBS) was purchased from Zhejiang Tianhang Biotechnology Co., Ltd. (Hangzhou, Zhejiang, China). Poly-L-Lysine was obtained from Solarbio (Beijing, China). Astragaloside IV (purity > 98%) was purchased from Yuanye Biotechnology Company Limited (Shanghai, China). The 3-methyladenineand rapamycin were provided by Sigma (St. Louis, MO, USA). The LDH assay kit was provided by Nanjing Jiancheng Bioengineering Institute (Nanjing, Jiangsu, China). CCK-8 was obtained from Beijing Zoman Biotechnology Co., Ltd. (Beijing, China). Antibody to Bcl-2 (ab692) was purchased from Abcam (Cambridge, MA, USA). Antibody to Bax (GB11007), antibody to P62 (GB11239-1), and antibody to LC3 (GB11124) were obtained from Servicebio (Wuhan, Hubei, China). Annexin V-FITC Apoptosis Detection Kit I was purchased from BD (BD Bio-Sciences Pharmingen, San Jose, CA, USA).

### 4.2. Middle Cerebral Artery Occlusion (MCAO)/Reperfusion (I/R) and Drug Treatment

A total of 18 adult Sprague Dawley (SD) rats (male; 280 ± 20) were provided by Beijing Vital River Laboratory Animal Technology Co., Ltd. (Permit No. SCXK (Beijing, China) 2016-0006) and randomly divided into three groups: Sham, MCAO, and Astragaloside IV + MCAO. All animals underwent middle cerebral artery occlusion/reperfusion except for the sham group. The SD rats were adapted for 1 week, fasted from food for 12 h, and fasted from water for 4 h. This was followed by intraperitoneal injection of 10% chloral hydrate. The anesthetized rats were placed in the supine position on operating table and shaved with sterilization around the incision.

The neck was dissected in the middle, and the left muscle was bluntly separated. The external carotid artery (ECA) was ligated and severed, and a suture was inserted from the ECA stump through the internal carotid artery (ICA) to reach the middle cerebral artery (MCA) to occlude the MCA [59]. The thread was pulled out gently to achieve reperfusion after two hours of ischemia; reperfusion lasted for 24 h. The rats in the sham group only had the left ICA and ECA exposed; they did not have MCAO. Astragaloside IV was injected intraperitoneal (i.p.) at 20 mg/kg during reperfusion [60]. All procedures followed the Guide for Care and Use of Laboratory Animals published by the National Institutes of Health. Hebei University of Chinese Medicine (Shijiazhuang, China) and the Animal Ethics Committee at this institution approved the protocol (approval number: HBTCM-2017-09; approval date: 20 September 2017).

### 4.3. Neurological Score Measurement

The neurological score was measured at 0 h after reperfusion and 24 h after reperfusion by an assessor who was unaware of the experiment groups. Zea Longa scoring: 0, normal performance, no neurological deficits; 1, contralateral forepaws cannot fully extend; 2, circling to the opposite side when walking; 3, falling to the opposite side when walking; 4, no spontaneous walking and loss of consciousness [61]. Of these, 1 to 3 points were listed as experimental subjects; 0 points, 4 points, and deaths (two rats died during middle cerebral artery occlusion surgery) were removed.

### 4.4. TTC Staining

Rats in each group were anesthetized, and the brains were collected and placed on ice for 24 h after reperfusion. Coronal thin slices (2 mm) of brain were placed into 2% TTC dye solution and incubated at 37 °C in the dark for 20 min. The staining solution was decanted, and TTC staining was terminated by rinsing with PBS, and the stained slices were photographed after fixation in 4% paraformaldehyde for 2 h. Unstained areas were defined as infarct, and the infarct volume and brain volume were calculated using Image-Pro Plus 6.0 image analysis software. The infarct volume (expressed as a percentage) was defined as total infarct volume/total brain volume × 100%.

### 4.5. Cell Culture

HT22 cells (neuronal line of mouse hippocampus) were from Professor Shun-jiang Xu and were cultured in complete culture medium (DMEM supplemented with 10% FBS). The cultures were maintained at 37 °C in a humidified atmosphere containing 5% CO_2_, and the media was changed every 2 days [62]. An in vitro model of I/R was built with these HT22 cells.

### 4.6. In Vitro Model of OGD/R

Oxygen and glucose deprivation/reoxygenation (OGD/R) is an accepted in vitro model for simulating I/R injury [63]. We used HT22 cells. First, the complete culture medium was discarded, and the cells were washed three times with PBS. Cells were added to glucose-free DMEM at 1% O_2_, 5% CO_2_, and 37 °C for 6 h. After the OGD period, the cells were washed three times with PBS, and complete culture medium was added. The cells were then maintained at 37 °C in a humidified atmosphere containing 5% CO_2_. Astragaloside IV was added to the HT22 cells at the same time as reoxygenation with continued culturing for 24 h (DMSO, the autophagy inhibitor 3ma (5 mmol/L), and the autophagy activator Rapa (250 nmol/L) were given similarly).

### 4.7. Cell Viability Analysis and Cytotoxicity Assay

CCK-8 was used to measure the viability of cells. The HT22 cells were cultured in 96-well plates (5 × 10^4^ cells/well) for 1 day and then treated with OGD/R. At the end of OGD/R, the HT22 cells were subjected to CCK-8. CCK-8 was added to HT22 cells (10 µL per well) for 1 h at 37 °C. The optical density was measured with a multi-function microplate reader (Varioskan LUX, Thermo, Waltham, MA, USA) at an emission of 450 nm. Cell viability was defined as (OD value of experimental group − OD value of blank group)/(OD value of control group − OD value of blank group) × 100%. An LDH assay was used to detect cytotoxicity. LDH will be released from cells when the cells are injured. The activity of LDH was measured at an emission of 450 nm. The LDH leakage rate was defined as LDH of cell supernatant/total LDH × 100%.

### 4.8. Western Blot

Cells were homogenized in lysis buffer containing phenylmethanesulfonyl fluoride (PMSF). The protein concentration was detected using a BCA method. The proteins were denatured and separated by 10% SDS polyacrylamide gel electrophoresis and transferred to polyvinylidene fluoride (PVDF) membrane via asemi-dry transfer method. PVDF membrane was blocked for 2 h using 5% fat milk, and incubated with an antibody to P62 (1:1000) and antibody to LC3 (1:1000) at 4 °C overnight. Primary antibodies were labeled with horseradish peroxidase-conjugated secondary antibody (1:3000). The Fusion FX5 Spectra Imaging System was used to obtained blot images directly from PVDF membrane, and the optical density of the bands was measured via Image J software (NIH, Bethesda, MD, USA).

### 4.9. Bax andBcl-2 Immunofluorescence Staining

Apoptosis was measured by Bax and Bcl-2 immunofluorescent staining. The HT22 cells were fixed with 4% paraformaldehyde for 20 min. The cells were permeabilized with 1% Triton X-100 for 15 min and then incubated in BSA for 30 min. Antibodies to Bax (1:100) and Bcl-2 (1:100) were added to cells, and the cells were incubated overnight at 4 °C. Primary antibodies were labeled with fluorescent secondary antibody. Cells were incubated with DAPI for 10 min in the dark. Each slide of the cells was randomly selected to take pictures of three fields-of-view and analyzed using Image Pro Plus 6.0 software (Media Cybernetics, Bethesda, MD, USA).

### 4.10. Flow Cytometry

Cells were dissociated by 0.25% trypsin (without EDTA), washed twice with PBS at 4 °C, centrifuged at 1500 r·min^−1^ for 5 min and re-suspended by 100 µL 1 × binding buffer. The 5 µL of FITC and 5 µL PI were added to cells after being re-suspended, and the cells were incubated in the dark for 15 min followed by 400 µL of 1 × binding buffer. The rate of apoptosis was measured by flow cytometry.

### 4.11. Data Analysis

All data were analyzed by SPSS 19.0 software (SPSS Inc., Chicago, IL, USA) and presented as the mean ± SD. One-way analysis of variance (ANOVA) was used on compare data from different groups. Tamhane’s T2 was used for post-hoc tests; *p* < 0.05 indicates statistically significant.

## 5. Conclusions

The data confirm that apoptosis and autophagy are fundamental to cerebral I/R injury, and increasing autophagy can protect neurons by down-regulating the level of apoptosis. Astragaloside IV can minimize cerebral injury in MCAO rats, increase the viability rates of HT22 cells after OGD/R, activate autophagy, and inhibit apoptosis. Astragaloside IV may exert neuroprotective effects by promotion of P62-LC3 autophagy to further inhibit apoptosis. These results have partially revealed the molecular neuroprotective mechanisms of Astragaloside IV.
